# Development of a Robust Multi-Scale Featured Local Binary Pattern for Improved Facial Expression Recognition

**DOI:** 10.3390/s20185391

**Published:** 2020-09-21

**Authors:** Suraiya Yasmin, Refat Khan Pathan, Munmun Biswas, Mayeen Uddin Khandaker, Mohammad Rashed Iqbal Faruque

**Affiliations:** 1 Department of Computer Science and Engineering, International Islamic University Chittagong, Chittagong-4318, Bangladesh; suraiyabrishti@gmail.com; 2 Department of Computer Science and Engineering, BGC Trust University Bangladesh, Chittagong-4381, Bangladesh; refatkhan93@gmail.com (R.K.P.); munmun@bgctub.ac.bd (M.B.); 3 Centre for Biomedical Physics, School of Healthcare and Medical Sciences, Sunway University, Bandar Sunway 47500, Selangor, Malaysia; 4 Space Science Centre (ANGKASA), Institute of Climate Change (IPI), Universiti Kebangsaan Malaysia, UKM, Bangi 43600, Selangor, Malaysia; rashed@ukm.edu.my

**Keywords:** facial expression recognition system, computer vision, multi-scale featured local binary pattern, unsharp masking, machine learning

## Abstract

Compelling facial expression recognition (FER) processes have been utilized in very successful fields like computer vision, robotics, artificial intelligence, and dynamic texture recognition. However, the FER’s critical problem with traditional local binary pattern (LBP) is the loss of neighboring pixels related to different scales that can affect the texture of facial images. To overcome such limitations, this study describes a new extended LBP method to extract feature vectors from images, detecting each image from facial expressions. The proposed method is based on the bitwise AND operation of two rotational kernels applied on LBP(8,1) and LBP(8,2) and utilizes two accessible datasets. Firstly, the facial parts are detected and the essential components of a face are observed, such as eyes, nose, and lips. The portion of the face is then cropped to reduce the dimensions and an unsharp masking kernel is applied to sharpen the image. The filtered images then go through the feature extraction method and wait for the classification process. Four machine learning classifiers were used to verify the proposed method. This study shows that the proposed multi-scale featured local binary pattern (MSFLBP), together with Support Vector Machine (SVM), outperformed the recent LBP-based state-of-the-art approaches resulting in an accuracy of 99.12% for the Extended Cohn–Kanade (CK+) dataset and 89.08% for the Karolinska Directed Emotional Faces (KDEF) dataset.

## 1. Introduction

Facial expression recognition (FER) is a regular and incredible sign to decipher the state of human feelings and expectations, expressing human emotion without saying anything, as faces are considerably more than key to singular personalities. In a word, one can say that it is one of the most natural, current, and robust means for communicating people’s intentions and emotions with others. As it is related to human emotion, which differs from one to another, researchers discovered many methods by both machine learning and deep learning techniques to obtain a critical understanding of this matter. Nowadays, things are becoming more mechanized through computer automation, where computer vision is playing a vital role in the automation process by training computers to interpret and understand the visual world. Thus, studies on FER show high demand in computer vision, which can be utilized in autonomy, neuro-advertising, scholastics, and altogether in security. Besides this, FER is one of the most challenging biometric recognition technologies due to its characteristics of nature, intuition, etc.

FER has two essential stages: feature extraction (geometric and appearance-based) and classification. While the geometrically-based feature extraction includes facial components like eye, mouth, nose, and eyebrow, the appearance-based one comprises the exact section of the face. On the other hand, the classification categorizes the expression, like a smile, sadness, anger, disgust, surprise, or fear. Researchers have worked with many neural networking concepts like Convolutional Neural Network (CNN), Recurrent Neural Network (RNN), and machine learning classifiers like Support Vector Machine (SVM), K Nearest Neighbour (KNN) to find, relatively, the most accurate FER technique. In connection to this, several researchers utilized the Neural Network based on different kinds of popular methods like CNN [1], CNN-RNN [2], 3DCNN-DAP [3,4], Weighted Mixture Deep Neural Network [5], CNN with attention mechanism (ACNN) where it empowers the model to move consideration from the impeded patches to other unhampered ones, just as distinct facial regions are dependent on patch-based ACNN (pACNN) and global-local based ACNN (gACNN) [6]. Although neural networks are easy to build with the latest programming languages like Python, R, and tools like Matlab and Weka, nevertheless, when it comes to the computational power, especially in facial image processing with many classes, it requires very high processing power with a high amount of random access memory (RAM) and a graphics processing unit (GPU). Additionally, suppose it is not a supercomputer. In that case, one needs hours to simply train a neural network model, which calculates too many features, where most of them are non-object-orientated, making the model prone to overfitting. However, since currently, the artificial intelligence (AI) receives a focal point to replicate or simulate human intelligence in machines, the incorporation of a multimodal concept (such as both machine learning and deep learning techniques) may produce a better FER compared to the typical models and sub-processes. 

Machine learning classifiers like SVM, KNN, and Tree cannot extract features automatically from raw images like the Neural Network (NN). Moreover, many other classifiers such as Principal Component Analysis (PCA), Extreme Learning Machine (ELM), Conditional Random Fields (CRF), and so on can also be used to classify facial emotion. However, classifiers need a state-of-the-art descriptor to extract a feature-set from natural images to classify into different classes. A wide range of methods and innovations have been tested by many researchers to find the best way for the classification of human disclosure. Features for FER are generally extracted with appearance-based methods like local binary pattern (LBP), local derivative pattern (LDP), local geometric binary (GLBP), and geometric methods like the histogram of oriented gradients (HOG), salient facial patches, classifier for salient areas on the faces [7], local binary pattern from three orthogonal planes (LBP-TOP) [8], local texture coding operator [9], and differential geometry. For instance, with the appearance-based method LBP, Zhang et al. applied a new method named Multi-resolution Histograms of Local Variation Patterns (MHLVP) on Gabor wavelets [10] and obtained a very impressive outcome on the Facial Recognition Technology (FERET) dataset; however, the computational complexity and element measurement was excessive. One of LBP’s universal drawbacks is relevant to its small 3 × 3 neighborhood, which cannot capture dominant features with large scale structures [11,12,13,14,15,16]. Zhao and Pietik Ainen extended the LBP operator to the spatiotemporal space, and they named it the volume local binary patterns model [17], which has been generally embraced in catching powerful features by rotating and concatenating different methods but worked with a single dataset, thus, the accuracy may fall for blurred images. Coming out from regular filtered images, features extraction with noise, and partial occlusions, a combined method of the histogram of oriented gradients (HOG) with the uniform-local ternary pattern (U-LTP) [18] is described, which gives a good filtering process as well. More discriminative features in higher-order derivative directions were captured by the LDP [19], which improved LBP. However, it is mostly limited to the surrounding eight-pixel values by avoiding more significant dimensional relations.

Along with LBP, many geometric based methods were also used in FER. Images are partitioned into blocks and sub-blocks, and an active appearance model was used for revealing the essential facial portions and extracted by differential geometric features [20] which has more accuracy in FER than the static geometric features, also provides valuable geometric data with the time and sequence of facial expression images. For non-formed images, a method of cases that were out-of-plane head revolutions was taken care of using the turn inversion invariant histogram of oriented gradients [21], which has insufficient time complexity and improved the learning model of the cascade to collaborate with the classification technique. Tsai and Chang have applied the filter of Gabor, discrete cosine, change, and transformation of angular radial [22] to use HFs, consolidating with self-quotient image (SQI) channels for improving FER accuracy under different light source environments. Typically, there are some miss images in the examination, and it is essential to include a non-face class in outward appearance classifications that are not clarified there. The facial illustration is to infer a gathering of features from unique face images to viably speaking faces. It should limit the inside class varieties of articulations while amplifying between class contrasts. In general circumstances, the geometric method needs very well structured facial images. Practically, most of the time, it is not possible to capture well-textured images to perform geometric methods. 

In addition to the many geometric and appearance-based methods, there are some more methods like the response method [23] that extracts features from directional texture and number patterns where performance is tested in constrained and unconstrained situations. Researchers have not been limited to static features only. There are some other methods for extracting dynamic and multilevel features [24], which have coordinated into an end-to-end network to participate flawlessly with one another. Moreover, to solve a small sample size (SSS) issue, using a novel method-directional multilinear independent component analysis (ICA) technique was demonstrated in [25], which prompts the dimensionality situation by encoding the input image or high dimensional data array as a general tensor. A different methodology for facial expression analysis is the use of the Human-Computer Interaction (HCI) context [26] disintegrated into smaller micro-decisions that are separately made by particular binary classifiers with higher accuracy of the general model. Besides the above-described methods, some methods are also used for the detection of real-time expressions such as embedded systems [27], Radon Barcodes [28], and many more. Classifiers acquire characteristic features from the above strategies as their sources as inputs. However, the classifier’s execution relies on the nature of feature vectors. A summary of a few recent works in the field of FER is shown in Table 1.

In light of the information mentioned above, one can observe a non-negligible limitation, especially in appearance-based typical LBP methods. Therefore, this study proposes a feature extraction method based on a new extended LBP “Multi-Scale Featured Local Binary Pattern”, which can be used not only in FER but also in various purposes to analyze an image. Since the automatic face expression recognition requires two significant angles: facial illustration and classifier style, this study utilizes four machine learning classifiers: SVM, KNN, Tree, and Discriminant Quadric Analysis. There are so many datasets, for example, Japanese Female Facial Expression (JAFFE), Chinese Academy of Sciences Institute of Automation (CASIA), Static Facial Expressions in the Wild (SFEW), Chinese Academy of Sciences Micro-expression-II (CASME), Spontaneous Micro-expression (SMIC), Acted Facial Expressions in the Wild (AFEW), and all are available in the literature. However, we used two well-known facial image datasets: Extended Cohn–Kanade Dataset (CK+) and Karolinska Directed Emotional Faces (KDEF) to verify our proposed method. Note that the Extended Cohn–Kanade Dataset (CK+) [29] is an extended version of Cohn–Kanade (CK) [30] and finds greater use in developing and evaluating facial expression analysis algorithms. It contains a better example of catching the sample space than the CK dataset, which includes 304 labeled videos with 5521 frames of test subjects from various ethnicities in varied age groups extending from 18 to 50.

**Figure 1:**
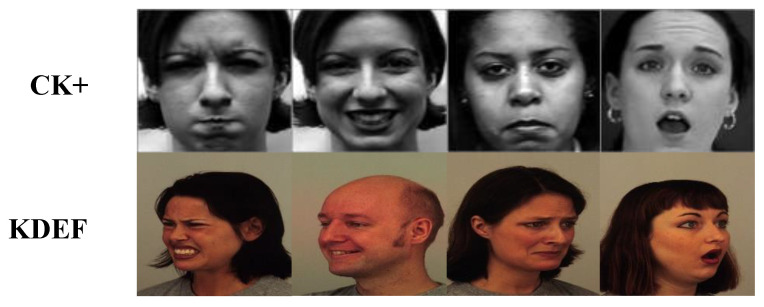
Sample face image from Extended Cohn–Kanade (CK+) and Karolinska Directed Emotional Faces (KDEF) datasets.

On the other hand, the used KDEF dataset helps assess the emotional contents and appraise intensity and arousal scale. Moreover, it contains a legitimate arrangement of feeling the full facial images. More details about these datasets are shown in Table 2 and some sample faces are shown in Figure 1.

## 2. Contribution

Based on the available literature, we observed that if the images are not well textured and blurred, then the prediction value falls. Thus, we have proposed a new feature extraction process for images that makes the texture of an image more machine-readable and converts the sub-region to 58 Uniform LBP and gives a classifier friendly feature vector tested on four machine learning classifiers. In this research, we have implemented three different angles where all the members are told to attempt to inspire the feeling that should have been expressed and to make the expression sharp and clear. The main contribution in the global LBP method is the process of calculating bitwise AND for two neighboring pixel values to obtain the relation between them after applying two suggested kernel matrices. Here, we have justified this method by detecting facial expression from an image that greatly relies on the image texture.

This manuscript is arranged with the proposed method in Section 3, including Section 3.1 pre-processing, Section 3.2: feature extraction, and Section 3.3: normalization. The result analysis is discussed in Section 4, and the conclusion is in Section 5.

## 3. Proposed Method

### 3.1. Pre-Processing

As the colored image sensitively affects light impact, the images were converted into grayscale as it has various shades of dark in the center, so to convert the image into grayscale, we used Equation (1) where *r* is the pixel value of red, *g* is green, and *b* is blue.

(1) gray=0.3r+0.59g+0.11b

**Figure 2:**
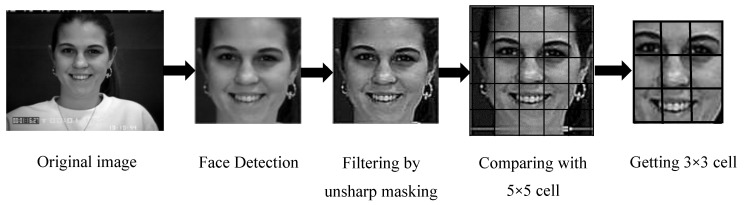
Pre-processing steps.

The grayscale image may have an environmental and useless background as well, which increases the computational complexity and misleading accuracy. From the CK+ and KDEF dataset of the raw image, it was observed that the images are size 640 × 490 and 562 × 762 pixels on average. Therefore, for better results and lower complexity, the facial part from the whole image was detected and the face was cropped by Haar cascade frontal face-based on the Viola-Jones detection algorithm, which precisely detects faces then crops and resizes them to 100 × 100 pixels. Each of the images was then compared with a 5 × 5 table cell and it was observed that key portions of models such as eyes, nose, and lips areas are in 3 × 3 table cells (60 × 60 pixels). Therefore, for avoiding the unnecessary parts, we have cropped this to 3 × 3 cells, shown in Figure 2. 

**Figure 3:**
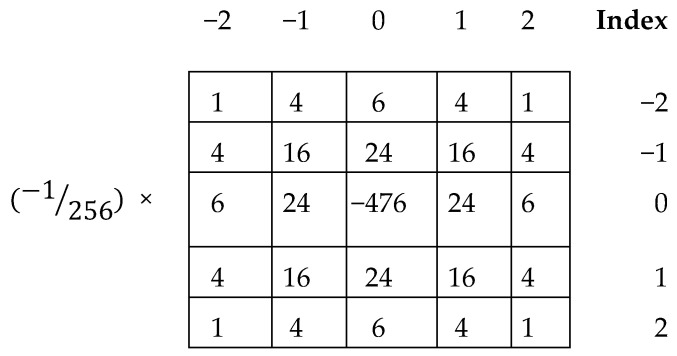
Unsharp masking kernel.

After detecting and cropping the images, the unsharp masking kernel [31] (shown in Figure 3) was used for sharpening the edges with Equation (2), which reduces some noises and gives a bright look. Grinding the images are essential for better understanding and communicating nearby grayscale change data by the contrast between each single points, and utilizes the weighted qualification in the eight directions as the local shade change data in the path, which is commotion and light-delicate and has no strength. The sharpening kernel was used in the side-by-side method where the Kernel moves in every one pixel.

(2) $$S\left(x,y\right)=\sum_{i=-2}^{2}\sum_{j=-2}^{2}K\left(i,j\right)\times M\left(x-i,y-j\right)$$

where *K* is the Kernel in Figure 3, and M is the pixel values of the given image, and *S*(*x*,*y*) is the central pixel value, which creates a sharpened image. The unsharp masking kernel was chosen in this study because it provides a good texture output in pixel values of different image datasets among many variants of kernels.

### 3.2. Feature Extraction

**Figure 4:**
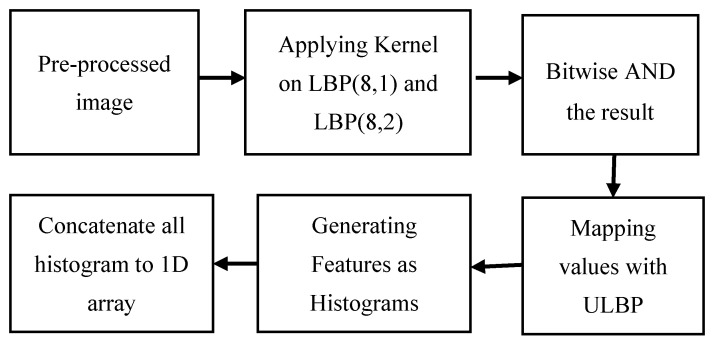
Feature extraction process.

**Figure 5:**
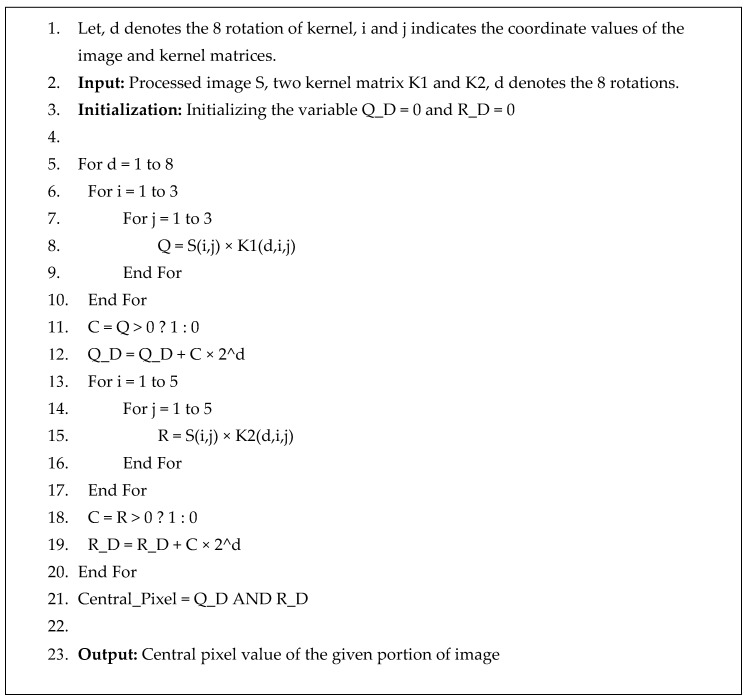
Feature extraction algorithm.

In this study, a method was developed for extracting features from an image to identify emotions. We depend not only on the shadow effect of the grayscale images but also on using a new kernel-based method to enhance the shadow effect to extract the features that are flexible and classifier friendly. We have proposed two kernels on the LBP of an image to be more precise about the shadow and light effect of the face parts, which mainly decides the face’s emotional states. In this step, the pre-processed image was taken and applied to the serial process shown in Figure 4 to finally obtain the features using the algorithm indicated in Figure 5.

**Figure 6:**
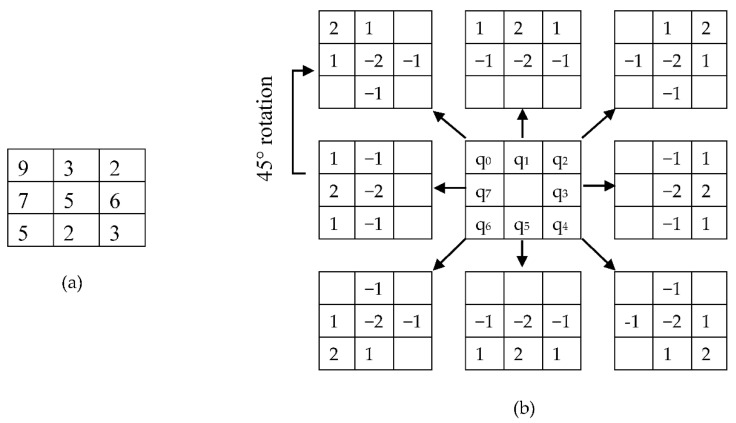
(**a**) Sample image segment of 3 × 3, (**b**) Description of Local Binary Pattern (LBP) _(8, 1)_: kernel value.

Generally, LBP _(P, R)_ is used in one radius on eight directional coordinates of the matrix value where P is the number of pixels to be considered and R is the radius from the central pixel. However, we used two LBP (LBP _(8, 1)_ and LBP _(8, 2)_) and applied two kernel matrix to calculate the central pixel of that cell. Considering the first stage of the image, we have divided it into sub-cells where 3 × 3 for LBP _(8, 1)_ and 5 × 5 for LBP _(8, 2)_ with two proposed kernels. A sample 3 × 3 image segment has been shown in Figure 6a and the model is shown in Figure 6b for the first Kernel, where each matrix is a 45° rotation, and the central matrix is the 3 × 3 cell of the pre-processed image. Considering that *S*_1_ denotes the grey estimation of the pixel point in the 3 × 3 neighborhood of the pre-processed image, and the kernel value of pixel points in the area is *K*_1_, the central pixel can be obtained by applying the first rotation kernel with Equation (3).

(3) $$G\left(x,y\right)=\sum_{i=-1}^{1}\sum_{j=-1}^{1}K_{1}\left(i,j\right)\times S_{1}\left(x-i,y-j\right)$$

**Figure 7:**
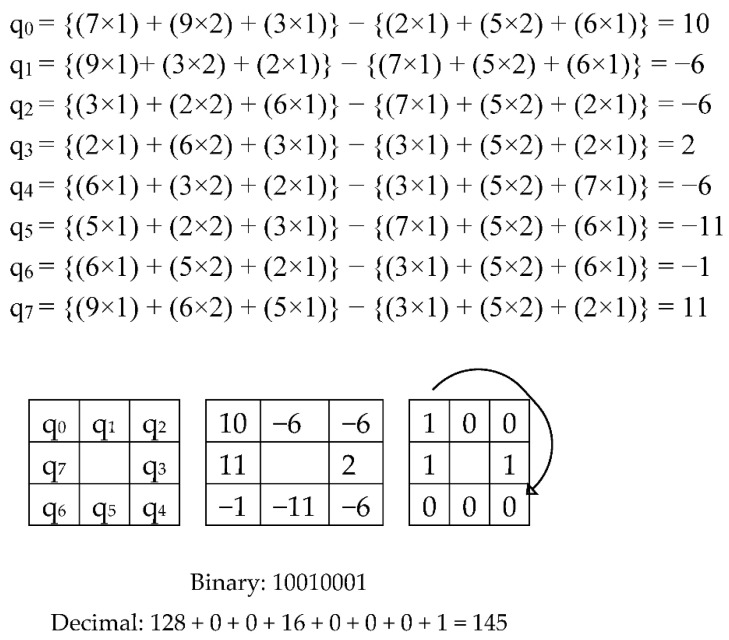
Calculation of LBP _(8, 1)_.

**Figure 8:**
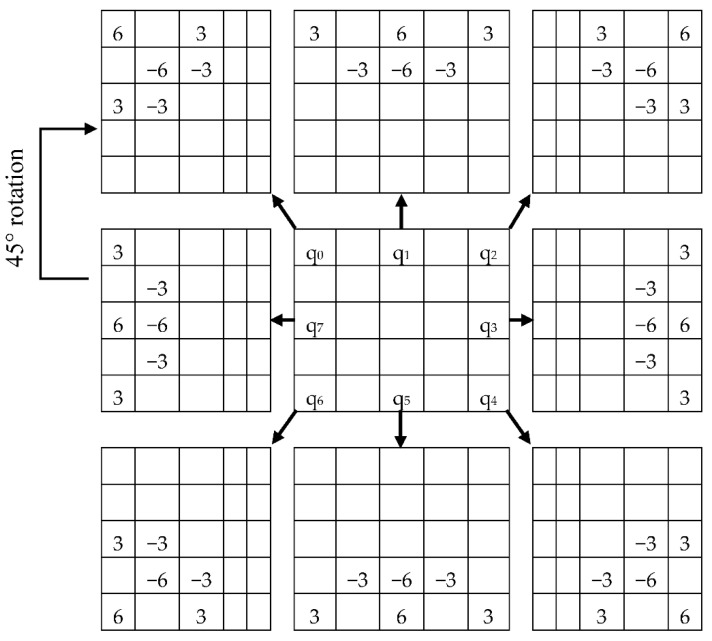
Description of LBP _(8, 2)_: kernel value.

**Figure 9:**
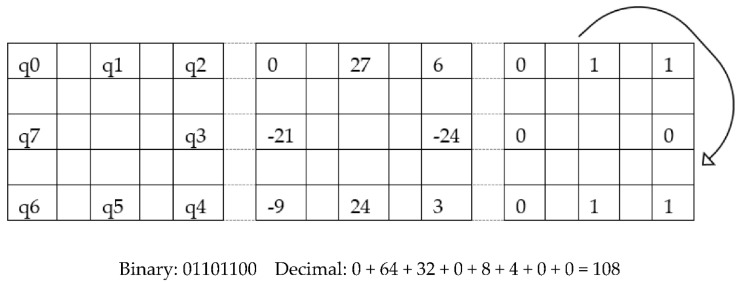
Calculation of LBP _(8, 2)_.

Here, K_1_ is eight rotational kernels with 45° rotations each. Therefore, Equation (3) was applied eight times to obtain the value q_0_ to q_7_ in Figure 7, G (x, y) is the central pixel value, which will make the pixel matrix for 1st Kernel. After the calculation is shown in Figure 7, converting the positive value as one and the negative value as 0, we obtain the central decimal pixel value. By using the sample image segment in Figure 6a, we used Equation (3) to show the calculation to find the central pixel matrix values q_0_ to q_7_ (as shown in Figure 7). This same procedure has been followed with the 5 × 5 image segment and kernel are shown in Figure 8 to find the central pixel matrix of Figure 9. 

The model for the second Kernel is shown in Figure 8, where each matrix is a 45° rotation, and the central matrix is 5 × 5 cells of the pre-processed image. Again, accepting that *S*_2_ denotes the grey estimation of the pixel point in the 5 × 5 neighborhood of the pre-processed image, and the kernel value of pixel points in the area is *K*_2_, the value of the central pixel can be obtained by applying the second Kernel with Equation (4).

(4) $$H\left(x,y\right)=\sum_{i=-2}^{2}\sum_{j=-2}^{2}K_{2}\left(i,j\right)\times S_{2}\left(x-i,y-j\right)$$

Similarly, kernel K_2_ will have eight rotations with 45° each for obtaining q_0_ to q_7_ values in Figure 9. *H* (*x*, *y*) is the central pixel which will make the pixel matrix for 2nd Kernel. Once again, converting the positive value as one and negative value as 0, we acquire the central decimal pixel value which is shown in Figure 9.

In the final stage, we have applied bitwise AND of *G* (*x*, *y*), *H* (*x*, *y*), where the binary output value of a model is determined to utilize Equation (5), which tells to the nearby change data between the center point and the 8-neighborhood pixels. It counts the number of spatial transitions from 0 to 1 or 1 to 0. In this stage, the equation will be as follows:

(5) $$BM\left(x,y\right)=(\sum_{i=-1}^{1}\sum_{j=-1}^{1}K_{1}\left(i,j\right)\times S_{1}\left(x-i,y-j\right))AND(\sum_{i=-2}^{2}\sum_{j=-2}^{2}K_{2}\left(i,j\right)\times S_{2}\left(x-i,y-j\right))$$

Simplifying Equation (5) as:

BM(x,y)=G(x,y) AND H(x,y)

where *BM* (*x*, *y*) is the binary matrix, the values of which are defined as 1 if *G* (*x*, *y*) = *H* (*x*, *y*) = 1 or 0 if any of *G* (*x*, *y*) or *H* (*x*, *y*) is 0.

We have used an assessment by applying a condition to find the output cell’s central pixel in decimal in Equation (6).

(6) $$MSLBP\left(x_{c,}y_{c}\right)=\sum_{n=0}^{7}BM(w_{n})2^{n}$$

where *w_n_* corresponds to the neighboring binary value of the eight surrounding pixels of the binary matrix *BM* and *MSLBP*(*x_c_*,*y_c_*) is the final central decimal pixel value. 

After calculating the *MSLBP* matrix, we have divided the whole image into 6 × 6 = 36 cells and mapped each cell’s value to the uniform local binary pattern (*ULBP*) by Equation (7). For *ULBP*, each cell pattern maps to 58-bin histograms. *ULBP* has unique 58 numbers where we will convert the *MSLBP* pixel matrix to a one-dimensional array by mapping pixel values to *ULBP* values. A single-cell value of 255 will be converted to 58 by using *ULBP*.

(7) $$FV=ULBP\left(MSLBP_{\left(x,y\right)}\right)$$

where *FV* is the feature vector, *ULBP* is the array of mapping values. *MSLBP* (*x*, *y*) is the pixel value of the image, which will be used as an index.

**Figure 10:**
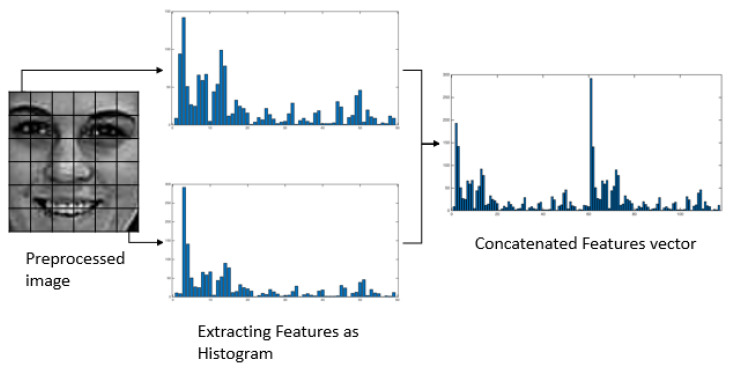
Converting process of selected geographical features of a histogram.

For one image, neighbor pixels are generally related; thus, the binary sequences of MSLBP (p, r) of the various radius can be seen as described. After ascertaining all values from left to right, we have obtained a binary pattern for every cell of an image. Taking all weighted values into account, we have found a decimal number in symmetric neighbor sets for various coordinates (x, y). The grey values of neighbors that are not the focal region for matrices can be evaluated by commitment. After that, we discovered one histogram for each cell, then we have concatenated all those histograms from each cell into a one-linear histogram shown in Figure 10. There will be a two-dimensional matrix for each image of seven classes where rows represent the image index, and the column represents the features. This long concatenated histogram is the initially featured vector with many noises and mismatched values within a class. We have normalized the histogram data to solve this kind of problem, which shows good accuracy in validation test cases compared with the original feature vectors. 

### 3.3. Normalization

**Figure 11:**
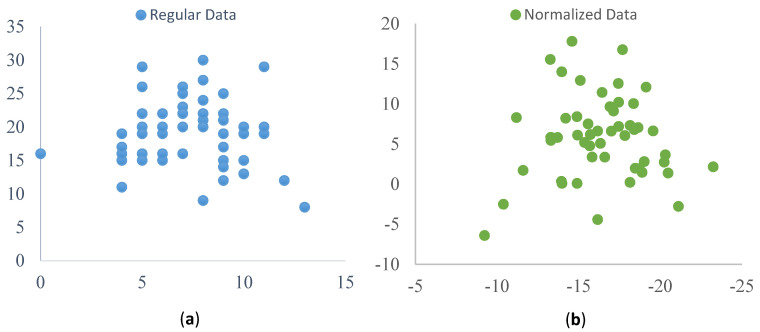
(**a**) Regular data, (**b**) Normalized data. Axis values are two feature values before (**a**) and after (**b**) normalization.

Due to the so many images with different expressions and features, it is challenging to maintain continuity among the classes. Therefore, normalization of data becomes mandatory to handle within a range of values so that each class keeps some kind of consistency. We have used the Generalized Procrustes Analysis (GPA) [32] as normalization in our proposed method. It takes each level data individually and utilizes a measure of variance. The GPA generates a weighting factor by analyzing the differences in the scaling factor applied to respondent scale usages and individual scale usage. As a result, the distance between different classes’ values was increased. Initially, we see the happy class’s data situated on the scatter plot shown in Figure 11a (before normalization), then we can see that the images are getting closer to each other in Figure 11b (after normalization). In brief, the GPA takes all those features and reduces the fluctuation, and after using this, all related emotional state values have become at a closer level which causes the classification to act more precisely as the variance increases between different classes. 

## 4. Results and Discussion

### 4.1. Performance Analysis of the Proposed Method

**Figure 12:**
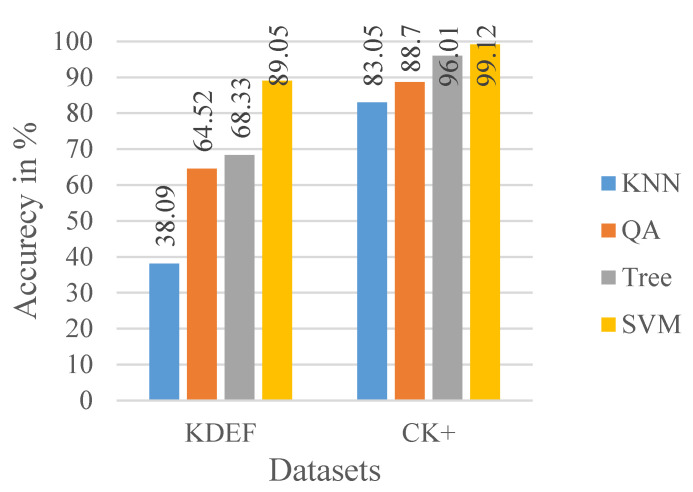
KDEF (KNN: 38.09, QA: 64.52, Tree: 68.33, SVM: 89.05), CK+ (KNN: 83.05, QA: 88.70, Tree: 96.01, SVM: 99.12).

We have tested our proposed method on the CK+ and KDEF dataset. The given datasets are the most widely used for facial expression recognition, and this includes seven different facial expression labels or classes. We have used several machine-learning classifiers like K-nearest neighbors (KNN), Binary Tree, Quadric Discriminant Analysis (QA), and Support Vector Machine (SVM) shown in Figure 12. Among them, SVM gives the highest testing accuracy, which is shown in the confusion matrix for both dataset’s test set following the 80-20 train-test split rule in Table 3 and Table 4, respectively. From the CK+ dataset, almost 6000 images are used for training and 2000 for validation and testing, and for the KDEF dataset, almost 2900 images are used for training and 1000 for validation and testing. A total of 10 iterations of K-Fold cross-validation was used in all four classifiers. All values are shown in percentage (%). 

The precision, recall, and F1 Score of the CK+ and KDEF dataset for SVM shows the outcome’s excellent structure. For finding these values, we first have to analyze the confusion matrix. When the actual class is positive, and the predicted class is also positive, it is counted as True Positive (TP) value. When the actual class is negative, and the predicted class is too negative, it is counted as a True Negative (TN) value. Along with these, if the actual class is positive but predicted as negative, it is counted as False Negative (FN). If the true class is negative but predicted as positive, it is counted as False Positive (FP).

Precision: It is the ration of *TP* and the total positive predictions. High precision means less classification error.

Precision=TP/(TP+FP)

Recall: It is the ration of *TP* and the total true positive classes.

Recall=TP/(TP+FN)

*F*1 Score: *F*1 Score is sometimes more useful than accuracy. It is the weighted average of the values of Precision and Recall. *F*1 Score is important here because we have an uneven number of classes.

F1 Score=2*(Precision* Recall)/(Precision + Recall)

**Figure 13:**
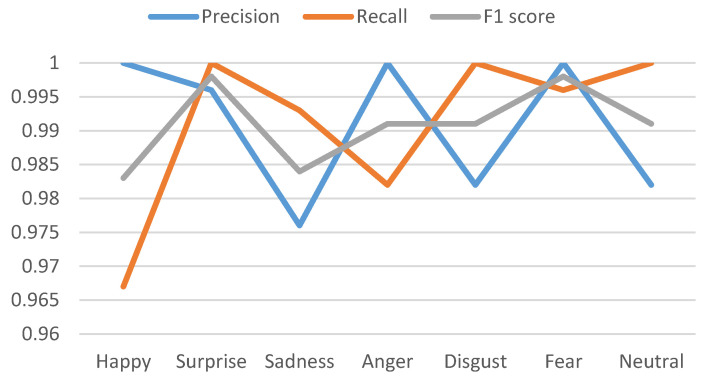
Dataset: CK+, Precision, Recall and F1 score shown for SVM.

**Figure 14:**
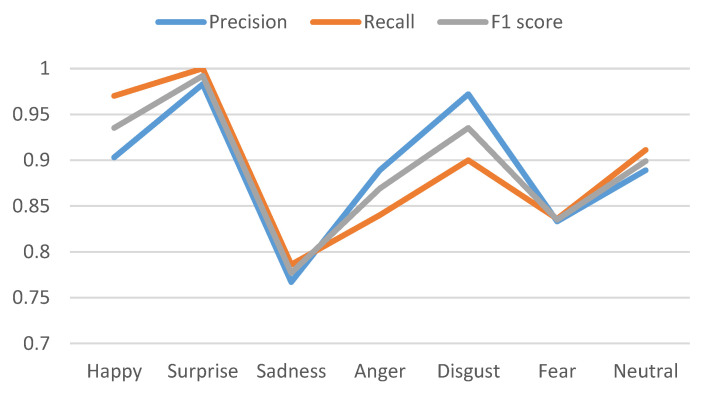
Dataset: KDEF, Precision, Recall, and F1 score is shown for SVM.

Table 5 shows the precision, recall, and *F*1 Score for datasets. We have presented the precision, recall, and *F*1 score comparatively in Figure 13 and Figure 14 for CK+ and KDEF datasets for all the K-folding cross-validations. Values are shown for SVM classifier because it has the highest accuracy.

### 4.2. Analyses and Discussion of Results 

Throughout this study, it is observed that classical LBP works with every pixel, which is contrasted and utilizes its eight surrounding 3 × 3 neighborhood by subtracting the center pixel value. Then, the resulting negative values are encoded with 0, otherwise 1. Finally, the encoded binary value is converted to decimal to obtain the center pixel value. The ongoing variety of LBP, for example, extended local binary patterns (ELBP) [15] operator not only performs the binary comparison of the center pixel and its neighbors but also encodes their exact grey-value differences (GDs) utilizing some extra binary units. In the completed modeling of the local binary pattern (CLBP) [16], it includes both the sign and the GDs between a given center pixel and its neighbors to improve the original LBP operator’s discriminative intensity. The two strategies have utilized LBP _(8,1)_ and compare the absolute value of GD with the given central pixel again to create an LBP-liked code. In Ref. [8], the authors first used the optical flow technique to obtain the Necessary Morphological Patches (NMPs) of micro-expressions; then, they calculated LBP-TOP operators by cascading them with optical flow histograms to make fusion features of dynamic patches. In local texture coding, the operator [9] enhances real-time system performance, utilizing four directional gradients on 5 × 5 grids for reducing sensitivity to noise. In Ref. [28], the authors present an observing framework using some features, such as LBP/LTP/red blood cell (RBC) for children, which utilizes an automatic pain detection system, and it could be accessed through wearable or mobile devices. A weighted fusion strategy [5] is proposed to completely utilize the features that were separated from various image channels with a partial Visual Geometry Group called the VGG16 network. Moreover, the method can develop consequently for extracting features of images on account of an absence of successful pre-prepared models dependent on LBP. The classical LBP and its varieties utilize pixel values of a different radius, but the relationships among them are missing. In this study, we have fulfilled the missing relational information among pixel values of varying radii. This study utilized an image into sub-cells where 3 × 3 for LBP _(8, 1)_ and 5 × 5 for LBP _(8, 2)_ with two proposed kernels with 45° rotations. After applying these kernels, bitwise AND operation occurred among the resulting matrices to establish the relation of different radii. Moreover, in pre-processing, we used the unsharp masking kernel to obtain a sharp image so that the intensity of pixel values can be more accurate. Compared with the neural network models, our method is a core algorithm to extract features where a neural network like CNN is a stack of automatic extraction of hidden layer features. Even though the latest neural network models are useful in the FER process, they still show unavoidable limitations. Different features like AAM/Arithmetic Unit system (AUs) [33] and Active Appearance Model (AAM)/Gabor [34] were used the CK+ dataset, and some other features like Gabor [35] and Facial Landmarks [36] used the KDEF datasets, all gaining different accuracies, which were much lower than our acquired accuracy. However, it can be expected that the addition of a neural network with our core algorithm to classify expressions might provide much higher efficiency on the other available standard FER datasets. Much readymade software, such as the Noldus network with Face-reader 8 [37] and Microsoft Emotion API [38], are available to obtain the facial expression easily from an image or live video. In Noldus face reader 8, besides FER, several things such as the detection of age, gender, ethnicity, facial hair, and glasses are performed. In doing so, a 3D model is created using the Active Appearance Method (AAM), and also an artificial neural network is used for training and classification. On the other hand, Microsoft Emotion API is a C# client-side library file, which is suitable for use as a third party API for detecting facial expressions in different projects under Microsoft Azure Cognitive Services. This API is licensed under the Massachusetts Institute of Technology (MIT), and the backend image processing model is developed and maintained by Microsoft. The primary comparison among Noldus Face-reader 8, Microsoft Emotion API and our work is incompatible as they are, in fact, software methods, and ours is a research method about MSFLBP. Moreover, only very little information is available on their methods, algorithms, and test results for building their FER models. 

The outcome of SVM on the proposed MSFLBP method is shown in Table 6, compared with some of the most recent state-of-the-art methods. It demonstrates that the proposed feature extraction method outperforms the most recent state-of-the-art methods. 

## 5. Conclusions

The study demonstrates the recognition rate improvement based on the calculation time of facial expression recognition methods. In the classification performance, we have used two notable datasets, CK+ and KDEF, and analyzed, as a set of cell size and number of direction, containers for the seven fundamental universal expressions’ exact characterization. We have used an unsharp masking kernel for sharpening the raw images. Then, we have applied two Kernel and bitwise AND to both binary matrices and converted the final binary matrix into a central decimal pixel value. After that, we have divided the output image into 64 cells and mapped each cell with ULBP mapping to obtain the features, like a histogram. By concatenating all cells’ assigned values, we have finally obtained the feature vector, which was then trained and tested with four classifiers with 10 K-Fold cross-validations. Among them, SVM provides the best outcome. In this study, the traditional LBP method’s limitations are overcome by applying bitwise AND on two rotational kernels by solving the pixel variance limitations. We have analyzed the neighboring pixel relation of traditional LBP and found two 3 × 3 and 5 × 5 kernels for obtaining the central pixel values, and after that, bitwise AND was applied to make the relation of the output central pixels of two kernels. Our described method can improve different texture recognition performance, utilize specific word applications with non-interrupting low-goals imaging, and also accomplish considerable accuracy. Several benefits of the described method include precise frequency extraction capability and less complexity, better efficiency in prediction, and fewer data storage. The addition of some more datasets from the different geographical regions can improve the real-time FER process. More combined methods like LBP-CNN can be used to identify augmented images.

## Figures and Tables

**Table 1:** Key information on some similar recently studied methods on facial expression recognition (FER).

Year	Classifier	Features	Databases
2015 [1]	SVM	CNN	FER/SFEW
2017 [5]	WMDNN	LBP	CK+/JAFFE/CASIA
2017 [7]	PCA	LBP/HOG	CK+/JAFFE
2019 [8]	SVM	LBP-TOP	CASME II/SMIC
2019 [9]	ELM	CS-LGC	CK+/JAFFE
2005 [10]	KNN	MHLVP	FERET
2007 [17]	SVM	VLBP/LBP-TOP	DynTex/MIT/CK+
2017 [18]	HOG	Ri-HOG	CK+/MMI/AFEW
2018 [20]	SVM	Differential Geometric Features	CK+
2017 [21]	HOG	Ri-HOG	CK+/MMI/AFEW
2017 [22]	SVM	FERS	CKFI/FG-NET/JAFFE
2019 [28]	SVM	LBP/LTP/RBC	Infant COPE

**Table 2:** Used datasets in the proposed method.

Dataset	No of Expressions Used	Image Size	No of Subject	Total Image
**CK+**	7	640 × 490	123	593 video sequence
**KDEF**	7	562 × 762	70	4900 Images

**Table 3:** Confusion matrix of the CK+ dataset (SVM).

	Happy	Surprise	Sadness	Anger	Disgust	Fear	Neutral
**Happy**	100	0	0	0	0	0	0
**Surprise**	0	99.67	0	0	0	0.33	0
**Sadness**	2.3256	0	97.67	0	0	0	0
**Anger**	0	0	0	100	0	0	0
**Disgust**	0	0	0	1.78	98.22	0	0
**Fear**	0	0	0	0	0	100	0
**Neutral**	1.04	0	0.68	0	0	0	98.28

**Table 4:** Confusion matrix of the KDEF dataset (SVM).

	Happy	Surprise	Sadness	Anger	Disgust	Fear	Neutral
**Happy**	90.28	0	0	0	9.72	0	0
**Surprise**	0	98.28	0	0	1.04	0	0.64
**Sadness**	0	0	76.72	8.56	0	9.44	5.28
**Anger**	0	0	4.17	88.89	0	4.17	2.78
**Disgust**	2.78	0	0	0	97.22	0	0
**Fear**	0	0	9.72	6.94	0	83.33	0
**Neutral**	0	0	6.94	1.39	0	2.78	88.89

**Table 5:** Pre (Precision), Rec (Recall), F1 (F1 Score) shown for dataset CK+, and KDEF. Values are shown for the Support Vector Machine (SVM) classifier for seven classes.

Classes	CK+			KDEF		
	Pre	Rec	F1	Pre	Rec	F1
**Happy**	1	0.967	0.983	0.903	0.970	0.935
**Surprise**	0.996	1	0.998	0.983	1	0.992
**Sadness**	0.976	0.993	0.984	0.767	0.786	0.777
**Anger**	1	0.982	0.991	0.889	0.840	0.869
**Disgust**	0.982	1	0.991	0.972	0.900	0.935
**Fear**	1	0.996	0.998	0.833	0.836	0.835
**Neutral**	0.982	1	0.991	0.889	0.911	0.899

**Table 6:** Results of reviewed works for static image approaches (values are in %).

Year	Classifier	Features	Databases	Accuracy (%)
2017 [5]	WMDNN	LBP	CK+/JAFFE/CASIA	97.02
2019 [8]	SVM	LBP-TOP	CASME II/SMIC	73.51/70.02
2019 [9]	ELM	CS-LGC	CK+/JAFFE	98.33/95.24
2017 [18]	HOG	Ri-HOG	CK+/MMI/AFEW	93.8/72.4/56.8
2019 [28]	SVM	LBP/LTP/RBC	Infant COPE	89.43/95.12
2016 [33]	SVM	AAM/AUs	CK+	54.47
2016 [36]	KNN	Landmarks	KDEF/JAFFE	92.29
2017 [34]	SVM/CRF	AAM/Gabor	CK+	93.93
2020	SVM	The proposed method (MSFLBP)	CK+	99.12
			KDEF	89.08
